# A Web-Based Training Intervention for Primary Care Providers on Preparing Patients for Cancer Treatment Decisions and Conversations About Clinical Trials: Evaluation of a Pilot Study Using Mixed Methods and Follow-Up

**DOI:** 10.2196/66892

**Published:** 2025-07-17

**Authors:** Naomi D Parker, Margo Michaels, Carla L Fisher, Alyssa Crowe, Elisa S Weiss, Maria Sae-Hau, Jason Arnold, Andrea Cassells, Domenic Durante, Ji-Hyun Lee, Raymond Mailhot Vega, Ana Natale-Pereira, Taylor S Vasquez, Zhongyue Zhang, Carma L Bylund

**Affiliations:** 1 Department of Health Outcomes and Biomedical Informatics College of Medicine University of Florida Gainesville, FL United States; 2 School of Public Health Boston University Boston, MA United States; 3 The Leukemia & Lymphoma Society Washington, DC United States; 4 Department of E-Learning, Technology, and Communications College of Education University of Florida Gainesville, FL United States; 5 Clinical Directors Network, Inc New York, NY United States; 6 Department of Biostatistics College of Public Health and Health Professions University of Florida Gainesville, FL United States; 7 Department of Radiation Oncology College of Medicine University of Florida Gainesville, FL United States; 8 Department of Medicine New Jersey Medical School Rutgers University Newark, NJ United States; 9 Department of Public Relations College of Journalism and Communications University of Florida Gainesville, FL United States; 10 UF Health Cancer Center University of Florida Gainesville, FL United States

**Keywords:** cancer clinical trials, continuing medical education, continuing education, primary care providers, provider-patient communication, cancer treatment, referral practices, online learning

## Abstract

**Background:**

Recruitment to cancer clinical trials (CCTs) is low, particularly for underrepresented groups such as uninsured patients, those with low-income status, and racial and ethnic minoritized individuals. A significant barrier is that treating oncologists often fail to inform patients about the possibility of CCT participation as an option for quality cancer care. Therefore, patient inquiries about trials before starting treatment should be normalized and encouraged, particularly for underrepresented groups. Primary care providers (PCPs) are uniquely suited to do this because they interact with patients at the time of cancer diagnosis, provide ongoing care, and are trusted sources of information.

**Objective:**

This study was designed to pilot an innovative web-based CCT training intervention for PCPs, including practicing clinicians and trainees, to increase their ability to prepare patients for cancer treatment decisions and conversations with oncologists about clinical trials.

**Methods:**

We conducted an evaluation of a pilot study using a self-guided, 1-hour web-based training intervention for PCPs with survey assessments before the intervention, immediately after the intervention, and at the 3-month follow-up. We used a mixed methods approach, incorporating quantitative and qualitative data collection and analysis. The evaluation was guided by the Kirkpatrick evaluation model, focusing on levels 1 (reaction), 2 (learning), and 3 (behavior).

**Results:**

A total of 29 PCPs completed the intervention and pre- and postintervention measures, with 28 (97%) PCPs completing the 3-month follow-up assessment. Of these 28 PCPs, 8 (29%) participated in a qualitative interview after the 3-month follow-up assessment. Participants reported high levels of satisfaction with the course. CCT knowledge, as well as attitudes and beliefs, improved after the course and were sustained at the 3-month follow-up. PCPs reported willingness to communicate with patients about cancer treatment options, including CCTs, and willingness to talk with their colleagues about potential changes in referral practices. However, fewer PCPs had actually engaged in these conversations by the 3-month follow-up. In the interviews, PCPs cited limited interprofessional knowledge sharing and organizational constraints as barriers. Notably, PCPs reported changes in their communication behavior with patients: a higher proportion reported communicating with patients at the time of referral about cancer treatment options and clinical trials at the 3-month follow-up than at baseline. In the interviews, PCPs reported that they felt more comfortable and empowered to have these conversations.

**Conclusions:**

This pilot study found that a self-guided, 1-hour web-based training intervention for PCPs resulted in improved knowledge, attitudes, and beliefs, as well as improved communication with patients, to prepare them for discussions with oncologists about cancer treatment and CCTs. Future dissemination of this course has the potential to make an impact on CCT accrual.

## Introduction

### Background

Cancer clinical trials (CCTs) are essential for advancing cancer treatment and ensuring quality of care for patients [1]. However, for cancer research to benefit all patients, it is critical to include participants who represent the diversity of the American population. Unfortunately, participation in cancer treatment trials remains low, with an overall accrual rate of only 7.1% [2]. Moreover, only approximately 5% of trial participants are Black or Hispanic, although these groups represent 15% and 13%, respectively, of people with cancer [3]. The ongoing underrepresentation of minoritized populations in CCTs not only raises concerns about equitable access to care but also limits the generalizability of the findings across diverse patient groups.

Various barriers at the system, institutional, clinician and research team, and patient levels contribute to low CCT enrollment [4-11]. One significant barrier is that a substantial portion of eligible patients are not approached about the possibility of receiving treatment through clinical trials [12]. This issue is especially pronounced for patients from demographic groups who are typically underrepresented in trials (eg, uninsured individuals, members of racial and ethnic minoritized groups, and people living in rural areas) [12-19] and who experience higher cancer mortality rates [20]. Even when the option of clinical trials is discussed with eligible patients, the communication is frequently unclear or inequitable, often reflecting racial disparities in how comprehensively information about CCTs is conveyed [21].

Research has shown that educating patients before their first oncologist visit can improve their knowledge, attitudes, and readiness for treatment decision-making, as well as increase their willingness to ask about and consider cancer treatment trials [22]. Therefore, it is important to normalize receiving treatment through clinical trials and encourage patient inquiries about trials before starting treatment, particularly for underrepresented groups. To effectively inquire about clinical trials, patients need to understand that quality care can be provided through CCTs, that asking about CCTs is encouraged and supported, that their participation in treatment decisions is encouraged [23,24], and that they are capable of fulfilling this role [8-10]. Patient education can start with the patient’s trusted primary care provider (PCP).

PCPs are a potential gateway facilitating access to CCTs because they interact with patients at the time of cancer diagnosis, provide ongoing care, and are trusted sources of information [25,26]. PCPs also report believing that they play an important role in patient care across the cancer continuum, including being actively involved during treatment [27]. Moreover, studies have shown that a trusted physician’s recommendation is a primary factor influencing patients’ decisions to enroll in clinical trials when offered [5,10,28-34] and that this influence may even be more acute for minoritized populations [35]. Therefore, improving PCPs’ attitudes, beliefs, and behaviors around CCTs may significantly influence patients’ attitudes and openness toward participation [36-39].

Although PCPs have expressed a desire for more of their patients with cancer to participate in CCTs, they often feel inadequately prepared to discuss trials due to their own limited understanding of CCTs as a high-quality treatment option [40,41]. In addition, they need guidance on effectively communicating with patients about potential trial participation. This gap in knowledge and skills presents a significant opportunity to educate PCPs about CCTs and their role in preparing patients for the oncology referral. These new skills can potentially improve trial accrual and patient access, especially for underrepresented groups.

Previous research, including our own, has demonstrated that conducting education with PCPs who serve underrepresented populations can contribute to breaking down key barriers to trial participation [42-44]. This can be achieved by improving PCPs’ capacity to both (1) educate recently diagnosed patients about the possibility of trial participation at the time of treatment referral to cancer care and (2) provide decision-making support to patients if advice is sought about trial participation after the patient has already met with the oncologist.

### Objectives

The purpose of this study was to pilot-test an innovative web-based training intervention for PCPs, including both practicing clinicians and trainees, designed to help them prepare patients for discussions about cancer treatment and CCTs. There are several types of CCTs, including prevention, screening, behavioral, and supportive care trials. However, for the purpose of this training, we focused specifically on treatment trials (eg, those involving chemotherapy or immunotherapy). We aimed to evaluate (1) PCPs’ general impressions of the training intervention and how the intervention impacted (2) their knowledge about CCTs, (3) their perceptions of their roles in caring for patients with cancer, (4) their approach toward and communication with patients, and (5) their willingness and behaviors in enacting practice-level changes.

## Methods

### Study Design

A 1-hour web-based training intervention for PCPs was developed and evaluated in a single-arm pilot study with assessments conducted before the intervention, immediately after the intervention, and at 3-month follow-up. We used a mixed methods approach, using quantitative and qualitative data collection and analysis to evaluate the training intervention [45]. We used the Kirkpatrick model to guide our evaluation [46]. The Kirkpatrick model proposes 4 sequential levels of training evaluation that become progressively more distal from the original training. In this study, we focused on levels 1 (reaction), 2 (learning), and 3 (behavior).

After conducting separate analyses, we integrated the results to allow the qualitative data to enhance and provide context for the quantitative data [47,48]. This approach integrates the data for triangulation, which strengthens the validity of the findings and allows for a more comprehensive understanding of the data [49-51]. This study followed the SQUIRE 2.0 (Standards for Quality Improvement Reporting Excellence) guidelines for assessing quality and reporting results [52].

### Training Intervention

We collaborated with instructional designers (DD and JA) from the University of Florida’s E-Learning, Technology, and Communications department to develop a 1-hour web-based training intervention for PCPs titled “Preparing Patients for Cancer Treatment Decisions: The Critical Role of Primary Care Providers in Facilitating Equitable Access to Care and Clinical Trials*.*” The content of the intervention was based on the authors’ collective research on the topic [40-43,53] as well as current literature on the role of PCPs in cancer treatment and referral, barriers to CCTs, patient activation, and best practices in medical education.

The asynchronous training intervention was hosted by a PCP (AN-P) and a radiation oncologist (RMV) who introduced content through engaging conversations and videos. Using a model of cognitive dissonance, the course introduced new information about CCTs, with the intent of helping providers realize inaccuracies in their knowledge, attitudes, and behaviors around CCTs and the referral to cancer treatment [54,55]. A clinical trials content expert (MM) additionally provided facts about CCTs, cancer care disparities, barriers to CCT participation, and the role of PCPs in cancer care. The 4 intervention modules covered content to enhance PCPs’ knowledge and communication skills surrounding cancer treatment discussions, including clinical trials and strengthening preparation related to the oncology referral process. The communication skills taught in the curriculum centered on the 5 E’s communication model (ie, explore, educate, encourage, engage in planning, and emphasize partnership), a framework designed to support patients in becoming more active participants in cancer treatment decision-making. Informed by principles of patient activation, treatment decision support, shared decision-making, and patient-centered communication, the model provides a simple mnemonic that clinicians can use to educate patients; prepare them for oncology referrals; and encourage inquiry about treatment options, including cancer treatment trials [56-59]. The learning objectives for each of the 4 modules are displayed in Table 1.

**Table 1:** Intervention modules and learning objectives.

Module	Learning focus	Learning objectives
1	Disparities in cancer care and clinical trial participation	List the reasons why cancer treatment trials are defined as quality cancer care Describe disparities in cancer care and in cancer treatment trial participation
2	Importance of strong PCP^a^ role in referrals to cancer treatment	Assess the critical role of the PCP in the referral process Assess the PCP’s role in preparing patients around treatment decision-making with the specialist
3	Communication skills to foster patient engagement in cancer treatment	Apply a 5-step communication approach to prepare patients for the specialist referral and their engagement in treatment planning
4	Needed changes with colleagues in the referral process	Evaluate the need to refine organizational procedures to improve one’s own referral practices for patients recently diagnosed with cancer

^a^PCP: primary care provider.

Throughout the modules, video vignettes featuring actors as patients and physicians further illustrate the 5E's communication model. The modules also include case studies where participants can explore patients’ disease histories, backgrounds, and cancer diagnoses. Participants complete each module at their own pace.

### Evaluation of the Training Intervention: Quantitative Assessments

#### Participants and Recruitment

We recruited PCPs to complete a web-based training intervention, pre- and postintervention surveys, and a postintervention interview. Recruitment was based on the following inclusion criteria: (1) being a PCP, including a physician (with MD or DO degrees), nurse practitioner (NP), physician assistant, or trainee (specifically postgraduate year [PGY] 2 or above in a family or internal medicine residency program) with at least 3 months of outpatient primary care experience; (2) working in an outpatient setting; (3) having made patient referrals to specialists for cancer treatment in the past year; and (4) speaking English and residing in the United States or its territories. Between July and December 2023, we emailed invitations that included a study description and instructions for those interested to contact a research coordinator. The invitations were distributed through email lists provided by three partner organizations: (1) the Clinical Directors Network, a national not-for-profit practice-based research network and clinician training organization; (2) Penn Medicine, an academic health system; and (3) the University of Florida’s Department of Community Health and Family Medicine and its family medicine and internal medicine residency programs, which are based in a large public academic health system. Once enrolled in the study, participants had up to 4 weeks to complete the training intervention.

#### Procedure

Participants completed a pretraining survey, an immediate posttraining survey, and a 3-month follow-up survey. Data were collected on the web via QualtricsXM (Qualtrics International Inc) [60]. The quantitative and qualitative phases of data collection and descriptions of each method are presented in Table 2.

**Table 2:** Descriptions of data collection measures and timeline.

Measures	Description	Before the training	After the training	3-mo follow-up
Participant characteristics	A 10-item survey that asks about participants’ demographics, professional background, and current professional setting	✓		
Knowledge of CCTs^a^	A 7-item measure comprising true or false statements about CCTs	✓	✓	✓
CCT attitudes and beliefs	A 4-item Likert-type measure that assesses attitudes, beliefs, and behavioral intentions related to CCTs	✓	✓	✓
Patient communication and referral practices	A 14-item measure that assesses the communication and referral behaviors of PCPs^b^ before (7 questions) and after (7 questions) returning patients’ initial referral to a cancer specialist to discuss treatment options	✓		✓
Willingness to change	A 3-item Likert-type measure that focuses on PCPs’ willingness to make changes in their practice	✓	✓	✓
Willingness to communicate	A 6-item Likert-type measure that focuses on PCPs’ willingness to engage with patients who have a cancer diagnosis	✓	✓	✓
Intervention usability	A 5-item survey that asks about participants’ device (eg, tablet computer), browser type, and whether they encountered any technical issues while taking the web-based course		✓	
Overall course satisfaction	A 10-item Likert-type measure where participants evaluate their training experience (eg, content and logistics) and intervention acceptability, along with 2 open-ended questions for additional feedback		✓	
Interview	A semistructured interview based on the quantitative survey measures, with an emphasis on exploring PCPs’ experiences with the course			✓

^a^CCT: cancer clinical trial.

^b^PCP: primary care provider.

Before beginning the training intervention and after providing informed consent, participants were asked to complete a 44-item web-based survey. The preintervention survey included 5 measures—knowledge of CCTs, CCT attitudes and beliefs, patient communication and referral practices, willingness to communicate, and willingness to change—all of which were informed by our previous work [40,41,43,53]. The preintervention survey also collected participant characteristics, including demographics and professional background. Immediately after the intervention, participants were asked to complete a 35-item web-based survey that included the knowledge of CCTs, CCT attitudes and beliefs, willingness to communicate, and willingness to change measures. In addition, they were asked about intervention usability and overall course satisfaction. Completing the intervention and the pre- and postintervention surveys fulfilled participants’ training requirements, at which point they were compensated US $150 for their participation.

Three months after the intervention, participants were asked to compete an additional 34-item web-based survey that included the knowledge of CCTs, CCT attitudes and beliefs, patient communication and referral practices, willingness to communicate, and willingness to change measures. After completing the 3-month follow-up survey, participants were compensated an additional US $45 for their participation. All surveys are shown in Multimedia Appendix 1.

#### Statistical Analysis

Descriptive statistics were used to analyze participants’ demographic characteristics. The data were analyzed using R software (version 4.4.0; R Foundation for Statistical Computing) [61]. Cronbach α values were used to measure the internal consistency among the CCT attitudes and beliefs, willingness to communicate, and willingness to change measures, and we report both individual item means and aggregated means for these measures. Frequencies (%) and mean (SD) scores for all survey measures were reported. The McNemar chi-square test and the Wilcoxon signed rank test were used to compare the results of the preintervention and postintervention surveys, including the 3-month follow-up survey. Due to the exploratory nature of this pilot study, adjustments for multiple comparisons were not made.

### Evaluation of the Training Intervention: Qualitative Phase

To gain further insights into the survey results, we conducted follow-up interviews with PCPs who completed the 3-month follow-up survey.

#### Participants and Recruitment

At the end of the 3-month follow-up survey, interested PCPs were invited to participate in a brief web-based interview to discuss their experience with the course. Between December 2023 and February 2024, members of the research team (A Crowe and NDP) contacted interested PCPs to confirm their participation and to schedule the interviews.

#### Interview Procedure

Between December 2023 and February 2024, two authors (NDP and A Crowe) interviewed PCPs via Zoom (Zoom Video Communications, Inc). The semistructured interview guide was designed based on the quantitative survey measures, with an emphasis on exploring PCPs’ experiences with the course. Participants were asked to share their impressions of the training intervention, their attitudes and beliefs regarding the role of PCPs, and to describe any changes they had made to their practice since taking the course. The interviews were audio recorded and professionally transcribed verbatim before analysis. Participants who completed the interview received an additional US $50 in compensation.

#### Qualitative Analysis

The interview data and analysis were managed using ATLAS.ti software (version 24.1.1; ATLAS.ti Scientific Software Development GmbH) [62]. Data collection and analysis were conducted concurrently, enabling an initial rapid analysis to capture and categorize data into broad themes [63,64]. Subsequently, a comprehensive thematic analysis was conducted by the first author (NDP), using both deductive and inductive coding strategies guided by the constant comparative method [65,66]. Throughout the analysis, 2 authors (NDP and CLF) met to review and discuss codes and emerging themes and to develop a codebook that guided the full thematic analysis [65,67]. Similar codes identified across all transcripts were collapsed into overarching themes, followed by axial coding to further characterize thematic properties [67].

### Data Integration

After conducting individual quantitative and qualitative analyses, we jointly interpreted the findings from both phases to enhance reporting. We report quantitative data first, which are then elaborated with the qualitative findings. When appropriate, we created joint displays to illustrate this data integration and to organize the results [48,68].

### Ethical Considerations

The study procedures were approved by the University of Florida Institutional Review Board (202200894). Before the PCPs participated in the surveys and interviews, they were provided a statement of participant rights and responsibilities and given the option to withdraw from the study at any time. All survey participants checked a box agreeing to participate before taking the survey. All interview participants verbally confirmed their willingness to participate, had the opportunity to ask questions before the interview, and were informed that interview data would be transcribed for analysis but only available to members of the research team. All data were deidentified before reporting in this study. Participants who completed the intervention and the pre- and postintervention surveys were compensated US $150. Participants who completed the 3-month follow-up survey were compensated an additional US $45.

## Results

### Overview

**Figure 1:**
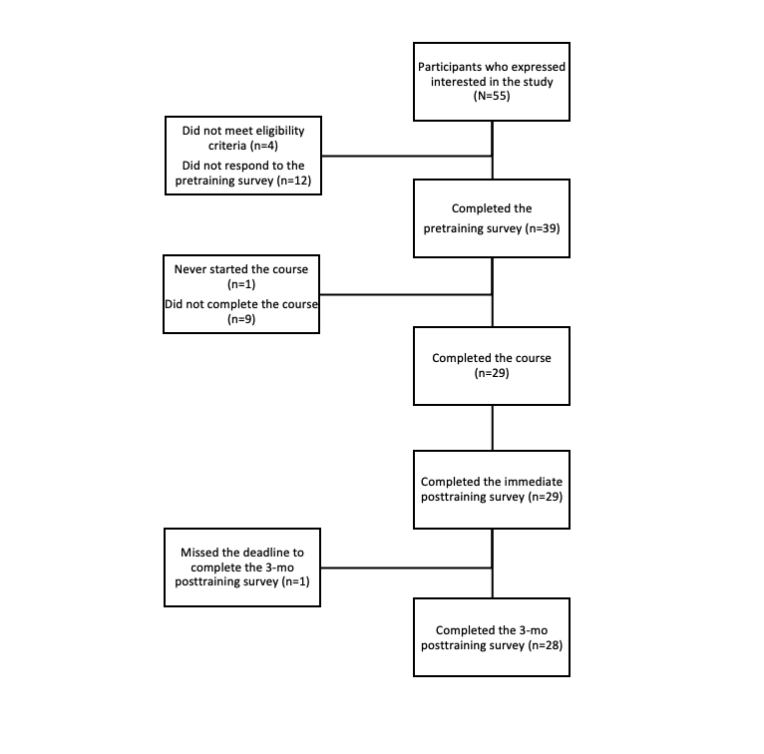
Flow diagram depicting study enrollment and participation.

A total of 29 PCPs completed the study and preintervention and postintervention surveys; the sample included 15 (52%) women and 14 (48%) men. Of the 29 PCPs, 28 (97%) completed the 3-month follow-up survey (Figure 1). Of these 28 participants, 8 (29%) took part in the interviews. Of the 29 participants, 26 (90%) had MD degrees, 2 (7%) were NPs, and 1 (3%) had a DO degree. More than half were physicians in training, with 17 (59%) describing themselves as residents or fellows in PGY2 (n=7, 41%), PGY3 (n=9, 53%), or PGY4 (n=1, 6%). Participants described their practice areas as internal medicine (16/29, 55%), family practice (11/29, 38%), or other (2/29, 7%). Most reported that their practice or training setting was an academic-affiliated hospital (17/29, 59%) or community-based and owned by an academic medical center (7/29, 24%). Complete demographic and professional characteristics of both survey and interview participants are shown in Table 3.

**Table 3:** Characteristics of surveyed and interviewed participants^a^.

Characteristics		Survey (n=29), n (%)	Interview (n=8), n (%)
**Sex**			
	Female	15 (52)	7 (88)
	Male	14 (48)	1 (12)
**Racial identity**			
	American Indian or Alaska Native	1 (3)	0 (0)
	Asian	9 (31)	4 (50)
	Black or African American	2 (7)	2 (25)
	White	13 (45)	2 (25)
	Multiracial	2 (7)	0 (0)
	Other	1 (3)	0 (0)
	Prefer not to respond	1 (3)	0 (0)
**Ethnicity**			
	Hispanic or Latino	1 (3)	0 (0)
	Not Hispanic or Latino	27 (93)	8 (100)
	Prefer not to respond	1 (3)	0 (0)
**Clinician type**			
	Doctor of medicine	26 (90)	7 (88)
	Nurse practitioner	2 (7)	1 (12)
	Doctor of osteopathic medicine	1 (3)	0 (0)
**Stage of training**			
	Resident or fellow	17 (59)	4 (50)
	Not resident or fellow	7 (24)	3 (38)
	Prefer not to respond	5 (17)	1 (12)
**Postgraduate year of training (if resident or fellow)**			
	2	7 (24)	1 (12)
	3	9 (31)	2 (25)
	4	1 (3)	1 (12)
	N/A^b^	12 (41)	0 (0)
	Missing	0 (0)	4 (50)
**Practice area**			
	Internal medicine	16 (55)	5 (62)
	Family practice	11 (38)	3 (38)
	Other	2 (7)	0 (0)
**Practice or training setting**			
	Academic-affiliated hospital	17 (59)	5 (62)
	Community-based, owned by an academic medical center	7 (24)	1 (12)
	Community-based, independent private	2 (7)	1 (12)
	Community hospital	2 (7)	1 (12)
	Community-based, owned by a large, nonhospital entity	1 (3)	0 (0)
**Is practice a designated federally qualified health center?**			
	Yes	15 (52)	4 (50)
	No	14 (48)	4 (50)
**Is practice supported by the Indian Health Service?**			
	No	27 (93)	8 (100)
	Yes	2 (7)	0 (0)

^a^Percentages may not add up to 100 because of rounding.

^b^N/A: not applicable.

The mixed methods findings were used to (1) characterize PCPs’ general impressions of the training intervention; in addition, we used these evaluation findings to capture how the training intervention impacted their (2) knowledge, (3) perceptions of their roles in caring for patients with cancer, (4) communication with their patients, and (5) practice-level change willingness and behaviors. The quantitative results are reported first and then elaborated upon with the qualitative findings within each of the 5 areas of impact.

### General Impressions of the Course

Both survey and interview findings illustrated PCPs’ positive impressions of, and high satisfaction with, the course. Specifically, PCPs expressed satisfaction with the course content, design and functionality, course elements, and course length. Their general impressions are presented in a joint display (Table 4) to depict the mixed methods results comprehensively.

**Table 4:** Primary care providers’ impressions of the course.

Course characteristics and quantitative measures			Survey question ratings^a^, mean (SD)		Illustrative quotes from the interviews
**Content**					
	“Course content was relevant to my work.”	4.6 (0.5)		“The content was excellent, and the way that it was provided was also very good. I found it very useful to learn information that I didn’t know before.” [P1]	
	“The course provided an adequate evidence base to support the content.”	4.5 (0.6)		“I thought it was kind of helpful...I’d never looked into discrepancies in cancer care. I probably could’ve suspected but didn’t know that was a fact.” [P8]	
	“The right amount of information was provided.”	4.6 (0.5)		“I felt [the course was] kind of straight to the point, and the topic was well laid out.” [P2]	
**Design and functionality**					
	“The course presented information in a clear and organized manner.”	4.7 (0.5)		“From an educational perspective, I thought it was designed pretty well. It was logical in the way that the modules flowed from one to the next.” [P3]	
	“The graphics (graphs, pictures, illustrations) added to the effectiveness of the presentation.”	4.5 (0.6)		“I thought [the course] was really well organized and laid out, and it had a nice, I guess, sequence of training.” [P4]	
**Course elements**					
	“The course scenarios facilitated my understanding.”	4.6 (0.5)		“I really enjoyed the videos, the examples, and the quiz questions...[and the] summary at the end, which I felt was helpful.” [P1]	
	“The interactive features in the course activities helped me learn.”	4.4 (0.8)		“I liked the different scenarios, and the short videos that you could engage with, and then immediately afterwards, trying to apply that to some questions.” [P3]	
	“The clinical interactions shown in the course felt authentic.”	4.3 (0.8)		“I really liked the case scenarios where it gave you different options for how you would respond to a patient. I thought that gave really valuable feedback in how we phrase some of the topics that we bring up.” [P4]	
	“The clinical interactions shown in the course were relatable to me.”	4.4 (0.6)		“I think, even as a primary care doctor who sees a lot of patients and who does diagnose cancer sometimes...It was really good content to push me to be more hands-on in my patients’ cancer care process.”‬ [P3]	
**Length of course**					
	“The length of the course was appropriate.”	4.7 (0.5)		“I thought that [the course] was educational and informative, and it was fairly straightforward and quick.” [P8]	

^a^Survey questions were scored on a 5-point scale ranging from 1=strongly disagree to 5=strongly agree.

### CCT Knowledge

PCPs’ positive feedback about the course aligns with their significant improvement in CCT knowledge after participating in the course. The survey results (Table 5) indicated that PCPs’ knowledge of CCTs was low before the training, with a mean score of 55% (SD 17.9%) across all 7 CCT knowledge items. Immediately after the training, PCPs’ knowledge of CCTs improved significantly to a mean score of 81% (SD 12.1%; *P*<.001). This improvement from before the training was sustained at the 3-month follow-up, with a mean score of 73% (SD 18.4%; *P*=.005).

**Table 5:** Comparison of correct responses on the pretraining and posttraining knowledge of cancer clinical trials (CCTs) survey^a^.

True or false CCT knowledge questions	Before the training (n=29), n (%)	After the training (n=29), n (%)	3-mo follow-up (n=28), n (%)	*P* value^b^ (before the training vs after the training)	*P* value^b^ (before the training vs 3-mo follow-up)
“In a cancer treatment trial, patients will receive a placebo alone or the new treatment being tested.”	19 (66)	26 (90)	21 (75)	*.046* ^c^	.70
“While about 25% of U.S. adults with cancer are eligible to participate in cancer treatment trials, only about 8% participate.”	20 (69)	29 (100)	27 (96)	*.008*	*.046*
“Patients determined to be eligible for cancer treatment trials are usually offered the opportunity to participate.”	14 (48)	23 (79)	22 (79)	*.04*	.06
“Patients from racial and ethnic minority groups eligible to participate in cancer treatment trials tend to be offered the opportunity to participate less frequently than are white patients.”	19 (66)	25 (86)	21 (75)	.15	.60
“When offered the opportunity to participate in a cancer treatment trial, patients from racial and ethnic minority groups agree to participate at about the same rate as do white patients.”	0 (0)	5 (17)	5 (18)	.07	.07
“Recommendation by a trusted physician is a primary factor influencing a patient’s decision to enroll in a cancer treatment trial.”	28 (97)	29 (100)	27 (96)	1	1
“Federal law now requires private insurers, Medicare and Medicaid to cover routine patient care costs in most cancer treatment trials.”	11 (38)	28 (97)	20 (71)	*<.001*	*.03*

^a^Percentage of aggregated correct responses: before the training, n=55 (18%); after the training, n=81 (12%); 3-mo follow-up, n=73 (18%); before the training versus after the training *P*<.001*;* before the training versus 3-mo follow-up *P*=*.*005.

^b^*P* values were calculated using the McNemar chi-square test with continuity correction, including only the participants who completed both pre- and posttraining surveys.

^c^Italicization indicates a statistically significant *P* value.

### PCPs’ Perceptions of Their Roles in Caring for Patients With Cancer

#### Overview

The findings demonstrated that the training intervention was effective in changing PCPs’ attitudes and beliefs about their role in caring for patients with cancer, which was sustained at the 3-month follow-up. As shown in Table 6, the scores across all 4 items increased significantly from before the training (mean 4.2, SD 0.6) to after the training (mean 4.6, SD 0.4; *P*<.001), as PCPs agreed that they play a role in their patients’ cancer care. This change was sustained at the 3-month follow-up (mean 4.5, SD 0.5; *P*=.004)*.* Most significantly, PCPs were more likely to agree that they “have an important role in educating patients about the possibility of receiving cancer treatment through a clinical trial, before the referral to a specialist” immediately after the training (mean 4.5, SD 0.6; *P*<.001) compared to before the training (mean 3.6, SD 1.0). This individual item improvement was also sustained at the 3-month follow-up (mean 4.4, SD 0.6; *P*<.001).

**Table 6:** Comparison of pretraining and posttraining scores on the cancer clinical trial attitudes and beliefs survey^a^.

Questions^b^	Before the training (n=29), mean (SD)	After the training (n=29), mean (SD)	3-mo follow-up (n=28), mean (SD)	*P* value^c^ (before the training vs after the training)	*P* value^c^ (before the training vs 3-mo follow-up)
“Health care providers like me have an important role in educating patients that there is often more than one option for treatment, before the referral to a specialist.”	4.2 (0.8)	4.6 (0.5)	4.5 (0.6)	*.008* ^d^	.07
“Health care providers like me have an important role in educating patients about the possibility of receiving cancer treatment through a clinical trial, before the referral to a specialist.”	3.6 (1.0)	4.5 (0.6)	4.4 (0.6)	*<.001*	*<.001*
“Health care providers like me have an important role in supporting patients’ decision to participate in a cancer treatment trial.”	4.4 (0.7)	4.7 (0.5)	4.6 (0.6)	.07	.30
“Health care providers like me can make a difference in the quality of cancer care our patients receive.”	4.6 (0.6)	4.7 (0.5)	4.5 (0.6)	.11	.80

^a^Mean of aggregated CCT attitudes and beliefs questions: before the training, mean 4.2 (SD 0.6); after the training, mean 4.6 (SD 0.4); 3-mo follow-up, mean 4.5 (SD 0.5); before the training versus after the training *P*<.001*;* before the training versus 3-mo follow-up *P*=*.*004. Survey questions were scored on a 5-point scale ranging from 1=strongly disagree to 5=strongly agree.

^b^Cronbach α value for the 4 CCT attitudes and beliefs questions was 0.82.

^c^*P* values were calculated using the Wilcoxon signed rank test with continuity correction, including only the participants who completed both pre- and posttraining surveys.

^d^Italicization indicates a statistically significant *P* value.

The qualitative findings shed light on these results by illustrating PCPs’ perceived role in caring for patients with cancer. After the 3-month follow-up, PCPs described fulfilling three key roles: (1) *partner*, (2) *educator*, and (3) *interpreter.* However, they also acknowledged struggling or feeling restricted in these roles due to *expertise boundaries*.

#### PCP as Partner

PCPs explained how they can fulfill roles beyond offering medical expertise by being partners. They emphasized the importance of *being a* *consistent source of support* by remaining involved in patients’ cancer journeys:

As a primary doctor, you really want to make your patient feel that you’re there. You’re not leaving...You still want to be a part of whatever plan is going to occur.

P2

Another PCP highlighted the collaborative nature of this ongoing support:

You have to look forward and make a plan, let [patients] know that they take one step, I take one step forward, and we’re kind of in tandem.

P7

PCPs also discussed *providing emotional support*, including helping patients manage their emotions and “the mental health issues that come along with [a] cancer diagnosis and cancer treatment, in general” (PCP1). Another PCP explained as follows:

When you initially tell the patient, they’re kind of like flabbergasted, and they’re in a state of shock...They’re just thinking “I’m going to die,” so I...try to help the patient get through the emotions, that initial shock.

P6

#### PCP as Educator

PCPs also discussed having roles as educators, where they focused on preparing patients for consultations with specialists by *discussing treatment options*, including introducing the possibility of receiving care through a CCT. PCPs emphasized being more proactive after the training intervention in ensuring that their patients were adequately informed to engage with specialists:

[The course] helped me to focus more on general treatment ideas, like radiation, surgery, chemotherapy, and sort of introduce them to those different treatment options so that then they could take that information and ask their oncologist and oncology surgeons about what is truly available to them, including clinical trials.

P4

A PCP additionally described *exploring patients’ understanding* of their treatment options, including clarifying misconceptions:

Whether it’s clinical trials or normal treatment, it’s important to explore [the patient’s] understanding of what’s going on, then you can kind of course correct, educate them on what actually might be happening and maybe where there’d be misconceptions.

P7

#### PCP as Interpreter

PCPs described taking on an interpreter role that centered on enhancing patients’ comprehension of medical information. PCPs described being a “go-between” (ie, a bridge) by acting as intermediaries who can interpret information provided to patients by oncologists and other cancer specialists:

[Patients] should come to me if they have questions, concerns, don’t understand something, because I am great at being a go-between in terms of reading notes and/or contacting oncologists, surgeons, interpreting things for them...and analyzing their options with them in ways that they understand.

P5

PCPs also recognized their role in *translating medical information*, such as information related to medications and laboratory results, to “kind of frame [the information] in terms the patient may know, just because some of the language isn’t as clear all the time” (PCP8). They recognized the value of making information more accessible for patients, although they were not cancer specialists themselves:

As a patient, it’s very overwhelming...but that’s kind of like why I’m here. I can try to interpret, maybe, better what’s going on...Like medications. What is it? What does it do? I mean, obviously, I’m not a specialist. I don’t know the ins and outs of everything, but I think kind of interpreting labs, a patient, a lot of times, they don’t [understand]...Just maybe a more kind of hands-on understanding for the patient.

P2

#### PCPs Navigating Expertise Boundaries

Finally, despite completing the training intervention, PCPs emphasized that they were still struggling with expertise boundaries. They admitted that their *lack of specialized knowledge* in oncology made them cautious about discussing cancer-related topics in detail:

I think the challenges as just a PCP...is that I just don’t have that knowledge of oncology, in specific. I don’t know enough about that specific clinical trial or that specific diagnosis, the prognosis, and things like that...Due to the lack of expertise, it makes it hard to communicate.

P1

PCPs further highlighted feeling the constraints of *role limitations* in guiding patients through complex cancer treatment decisions:

Oftentimes the questions [that patients are] asking about the treatments are very nuanced...I think there’s only so much that the primary care doctor can answer.

P7

### Communication About Treatment Options

#### Overview

The survey findings demonstrated that the training intervention enhanced PCPs’ *willingness to communicate* with their patients about cancer treatment options in general, including CCTs (Table 7). Mean scores across the 6 items increased significantly from before the training (mean 4.4, SD 0.5) to immediately after the training (mean 4.7, SD 0.5; *P*=.007). This improvement was not sustained at the 3-month follow-up. However, 1 item—PCPs’ willingness to “educate these patients about the possibility of receiving treatment within a cancer clinical trial”—showed significant improvement at the 3-month follow-up (mean 4.4, SD 0.6; *P*=.04) compared to before the training (mean 4.0, SD 0.8).

**Table 7:** Comparison of pretraining and posttraining scores on the willingness to communicate survey^a^.

Questions^b^	Before the training (n=29), mean (SD)	After the training (n=29), mean (SD)	3-mo follow-up (n=28), mean (SD)	*P* value^c^ (before the training vs after the training)	*P* value^c^ (before the training vs 3-mo follow-up)
“I am willing to explore patients’ concerns about cancer treatment.”	4.4 (0.5)	4.7 (0.5)	4.5 (0.5)	*.01* ^d^	.60
“I am willing to educate these patients that there is often more than one option for treatment.”	4.4 (0.7)	4.7 (0.6)	4.5 (0.6)	*.04*	.70
“I am willing to educate these patients about the possibility of receiving treatment within a cancer clinical trial.”	4.0 (0.8)	4.6 (0.6)	4.4 (0.6)	*.003*	*.04*
“I am willing to encourage these patients to ask questions of the specialist about different treatment options.”	4.6 (0.6)	4.7 (0.5)	4.4 (0.7)	.30	.30
“I am willing to encourage these patients to ask questions of the specialist about receiving treatment within a cancer clinical trial.”	4.4 (0.6)	4.7 (0.5)	4.5 (0.5)	.14	.60
“I am willing to emphasize my role as a partner throughout these patients’ cancer care.”	4.6 (0.5)	4.7 (0.5)	4.6 (0.6)	.20	.80
Mean of aggregated willingness to communicate questions	4.4 (0.5)	4.7 (0.5)	4.5 (0.5)	*.007*	.50

^a^Mean of aggregated willingness to communicate questions: before the training, mean 4.4 (SD 0.5); after the training, mean 4.7 (SD 0.5); 3-mo follow-up, mean 4.5 (SD 0.5); before the training versus after the training *P*=.007*;* before the training versus 3-mo follow-up *P*=*.*50. Survey questions were scored on a 5-point scale ranging from 1=*strongly disagree* to 5=*strongly agree*.

^b^Cronbach α value for the 6 willingness to communicate questions was 0.91.

^c^*P* values were calculated using the Wilcoxon signed rank test with continuity correction, including only the participants who completed both pre- and posttraining surveys.

^d^Italicization indicates a statistically significant *P* value.

The survey results also showed that the training intervention influenced PCPs’ self-reported *patient communication and referral practices* immediately after the training and at the 3-month follow-up. As shown in Table 8, seven items on the patient communication and referral practices measure focused on the 5Es model from the curriculum (ie, explore, educate, encourage, engage in planning, and emphasize partnership). We asked PCPs about their communication in two different contexts of seeing patients: (1) before making an initial patient referral and (2) after patients returned following their referral appointment. After the training, PCPs reported improvements in both contexts. Specifically, they reported discussing cancer treatment options with a higher percentage of patients in the “before making a referral” context (ie, context 1). Overall, 6 (86%) of the 7 items showed improvement, with the most significant changes seen on the 2 items focusing specifically on CCTs. PCPs reported educating a higher percentage of patients “about receiving treatment with a cancer clinical trial” 3 months after completing the training intervention (before the training: mean 15%, SD 20.2%; 3-mo follow-up: mean 48%, SD 30.5%; *P*<.001). They also reported educating a higher percentage of patients to “encourage inquiry about receiving treatment through a cancer clinical trial” (before the training: mean 22%, SD 29.4%; 3-mo follow-up: mean 62%, SD 34.9%; *P*<.001). When surveyed about their behaviors after patients returned following their referral appointment (ie, context 2), these same 2 items were the only ones that showed significant improvement.

**Table 8:** Comparison of pretraining and 3-month follow-up scores on the patient communication and referral practices survey.

Questions (regarding patients diagnosed with cancer in the past 3 mo)		Before the training (n=29), mean (SD)	3-mo follow-up (n=28), mean (SD)	*P* value^a^	Illustrative quotes
“**Prior to making a referral, approx. what percentage of patients did you:”^b^**					
	“Explore concerns about cancer treatment in general”	59.8 (35.9)	75.4 (27.1)	*.03* ^c^	“[The course] reaffirmed the importance of leveraging the primary care relationship...A lot of my patients circle back to ask about my opinions before starting a specific type of cancer treatment.” [P3]
	“Educate about cancer treatment in general”	54.8 (32.7)	73.4 (34.2)	*.006*	“[Patients are] meeting these oncologists for the first time and...these are people I’ve known for years, so it makes sense that I also still have a role in making sure that they understand clinical trials, but also the treatment options that have been made available to them by the oncologist.” [P7]
	“Educate about receiving treatment with a cancer clinical trial”	15.2 (20.2)	47.9 (30.5)	*<.001*	“[Before the course] I didn’t realize that PCPs can also play a role and influence patient decisions [about clinical trials].” [P1]
	“Encourage inquiry about treatment options”	68.8 (37.6)	83.8 (28.0)	*.04*	“The important role of the PCP, that was interesting to me. I thought...the oncologist probably had more impact. But I can see that if you have a good relationship with your patient, then you can also encourage them one way or the other, or maybe as a second opinion, to help them figure out what they want for their treatment plan.” [P1]
	“Encourage inquiry about receiving treatment through a cancer clinical trial”	21.7 (29.4)	61.6 (34.9)	*<.001*	“[I now remember] to emphasize and mention the possibility of clinical trials when they see their specialists, particularly when I'm working with minority patients to...remind them to consider [clinical trials] and to ask” [P5]
	“Engage in helping patients plan these inquiries with a specialist”	48.3 (37.2)	70.2 (30.2)	*.007*	“Putting on people’s radars...the availability of trials and asking patients to talk to specialists about the potential to participate in trial was valuable.” [P5]
	“Emphasize partnership as patients go through cancer care”	80.7 (32.6)	83.6 (29.4)	.60	“I kind of felt like I needed to do a better job on being more intentional about the follow-ups...Maybe having more check-ins to see how the treatment is going, how their mental health is doing and stuff like that is important. So that was one thing I’ve been trying to do is schedule more follow-ups during their active cancer treatment.” [P3]
“**After patients returned following their initial referral, approx. what percentage did you:”**^b^					
	“Explore concerns about cancer treatment in general”	62.3 (38.6)	53.4 (44.3)	.40	“What I’ve noticed is a patient will get diagnosed with something...then they’ll come to us as a primary care doctor and sort of ask questions, like, they want us to help guide them through the decision-making process, which is great.” [P7]
	“Educate about cancer treatment in general”	56.9 (35.0)	58.1 (42.6)	.60	“In a couple of cases [my patient] point blank said, ‘You’ve explained my diagnosis and my treatment options better than the other people that I talked to...I felt very prepared with asking [my oncologist] about specific treatment options.’” [P4]
	“Educate about receiving treatment with a cancer clinical trial”	23.3 (32.4)	50.4 (40.7)	*.003*	“[A takeaway for me was] the involvement of a PCP in helping make some of those decisions and helping patients feel open to [cancer clinical trials]. I do get the sense of encouraging the patient or empowering them a little bit and supporting them in terms of whatever their [treatment] decision might be. It was helpful to see that there is a true role for that.” [P8]
	“Encourage inquiry about treatment options”	59.6 (37.8)	64.4 (41.5)	.70	“I think...just presenting the different treatment options for the patients really got them to feel more comfortable asking questions when they were ready to see their oncologist.” [P4]
	“Encourage inquiry about receiving treatment through a cancer clinical trial”	19.6 (25.9)	55.9 (43.1)	*<.001*	“I actually encourage [my patients] to ask more treatment discussion and decision-making questions with their oncologist. I just explain to them that, you should write [questions] down in ways that they'll remember, right?” [P7]
	“Engage in helping patients plan these inquiries with a specialist”	47.8 (40.2)	61.0 (42.7)	.13	“I try to use that connection and that existing relationship to help patients feel comfortable in the decisions they’re making and then also to support the specialists in our academic institution to try to help them also build confidence in the new specialists that they’re seeing.” [P3]
	“Emphasize partnership as patients go through cancer care”	73.2 (36.0)	69.5 (41.4)	.70	“I think what stood out to me is there’s an emphasis on making sure patients understand their diagnosis...and the importance of following up...and [that] the oncologist is meeting them probably for the first or second time when you’re talking about clinical trials and cancer treatments.” [P7]

^a^*P* values were calculated using the Wilcoxon signed rank test with continuity correction, including only the participants who completed both pre- and posttraining surveys.

^b^Each question was answered as a percentage, ranging from 0 to 100.

^c^Italicization indicates a statistically significant *P* value.

The findings from the interviews expand on these results by illustrating how PCPs’ approach to patient communication evolved after the course. Collectively, they reported an increased sense of self-efficacy, feeling more comfortable and empowered in discussing cancer treatment options with patients. PCPs emphasized the importance of three goals in their approach: (1) engage patients, (2) empower patients, and (3) promote equity. PCPs described using specific communication approaches to achieve each of these goals.

#### Engage Patients

First, PCPs described a shift in practice after taking the course, working to engage patients in informed discussions about treatment options, including CCTs, rather than deferring these conversations to oncologists. They emphasized taking a proactive approach by initiating discussions about treatment options:

[The course] helped me to be more aware [of treatment options], so I can at least have that conversation with the patient.

P2

[The course] certainly opened my eyes to the fact that [treatment options] should be mentioned, something I would not have previously done.

P8

PCPs also highlighted encouraging consideration of clinical trials when communicating with patients about their cancer treatment options:

After the course, I’m just more likely to bring up clinical trials, whether there’s a clinical trial [available] or not.

P7

My approach, it changed a little bit, to the point that I’m more willing to talk to [patients] a little bit more about [CCTs], rather than just immediately just defer everything to oncology.

P1

#### Empower Patients

Next, PCPs highlighted their efforts in empowering patients to “take ownership of their own health care” (PCP4). PCPs discussed prompting patients to thoroughly *explore treatment options*, ensuring that they are aware of the possibilities available to them:

I...either encourage them to at least take a look at what that [treatment] option is for them or encourage them to deep-dive into that option.

P1

Moreover, PCPs emphasized encouraging their patients to *ask specialists questions about treatment options*:

[I tell patients] “I can answer as many questions as I can, but the ones that I can’t...you can go back to the oncologist and ask them.” So, I encourage them to ask more treatment discussion and decision-making questions with their oncologist.

P7

#### Promote Equity

Finally, PCPs discussed being more aware of the importance of promoting equity when discussing cancer treatment options. PCPs described *tailoring patient communication* to incorporate patients’ diverse needs and to actively engage those who are part of groups who are underrepresented in clinical trials:

[Before the course] I didn’t realize...that there is a discrepancy on, for example, race, and those that participate in clinical trials...When I see that patient, I now know that I should actively engage them and talk to them about clinical trials and prime them before they go in to see the oncologist, for example...If you know where...the lack of utilization is, you can kind of intervene and make it more of an active conversation with the patients.

P7

PCPs also highlighted being “far more cognizant” (PCP5) after the course about *facilitating patients’ access* to treatment options to ensure “patients are getting offered the same [care] as everyone else” (PCP2). A PCP emphasized as follows:

I was appreciative that the [course] modules focused on [disparities in CCT participation], and I am trying to be more equitable in the care that I provide to my patients as well, by being sure that I offer all the treatment options...You never know how much that could change someone’s life.

P4

### PCPs’ Practice-Level Changes

#### Overview

We also examined PCPs’ willingness or ability to enact practice-level changes when referring patients to cancer specialists (Table 9). At the posttest assessment, PCPs reported high levels of willingness to enact these changes (mean 4.3, SD 0.6). However, at the 3-month follow-up, PCPs reported lower levels of agreement, although still above midpoint, with statements indicating that they had had these conversations (mean 3.7, SD 0.8). Notably, although 24 (83%) of the 29 participating PCPs completed the course’s interactive action plan, none of those interviewed reported printing the plan for future reference, which was provided as a direction in the course. Of the 8 PCPs who were interviewed, 5 (63%) stated that they did not have access to a printer, 2 (25%) mentioned being too busy, and 1 (12%) did not provide an answer.

**Table 9:** Comparison of pretraining and posttraining and 3-month follow-up scores on the willingness to change survey^a^.

Assessments and questions^b^			Scores, mean (SD)
**Posttraining survey (n=29)^c^**			
	“I am willing to make needed changes to whom I refer my patients for cancer care, in order to improve their access to cancer treatment trials.”	4.3 (0.7)	
	“I am willing to talk with colleagues in my practice about needed changes in how we educate our patients with cancer prior to referral.”	4.3 (0.7)	
	“I am willing to talk with colleagues in my practice about needed changes to whom we refer our patients for cancer care, in order to improve their access to cancer treatment trials.”	4.3 (0.6)	
**3-mo follow-up survey (n=28)^d^**			
	“I have made changes to whom I refer my patients for cancer care, in order to improve their access to cancer treatment trials.”	3.9 (1.0)	
	“I have talked with colleagues in my practice about needed changes in how we educate our patients with cancer prior to referral.”	3.7 (1.0)	
	“I have talked with colleagues in my practice about needed changes to whom we refer our patients for cancer care, in order to improve their access to cancer treatment trials.”	3.5 (0.9)	

^a^Survey questions were scored on a 5-point scale ranging from 1=strongly disagree to 5=strongly agree.

^b^Cronbach α values for the 3 willingness to change questions was 0.9.

^c^Mean of aggregated willingness to change questions=4.3 (SD 0.6).

^d^Mean of aggregated willingness to change questions=3.7 (SD 0.8).

The qualitative findings further illuminate the underlying factors contributing to PCPs’ hesitancy or unwillingness to implement practice-level changes. Most PCPs reported facing systemic barriers, specifically highlighting (1) *limited interprofessional knowledge sharing* and (2) *organizational constraints*.

#### Limited Interprofessional Knowledge Sharing

First, PCPs cited how limited interprofessional knowledge sharing within their practice environments was a barrier to discussing and enacting change for improving cancer care and referral practices. Instead, PCPs shared, “we kind of just do our own thing” (PCP2). PCPs emphasized that a *lack of collaboration opportunities* made informal or formal discussions within their practice settings scarce:

Part of the course involved talking with colleagues about best ways to advocate for our patients and potentially system-change stuff for advising patients about clinical trials...Loved the idea, but...We typically work at our individual desks through lunch and rarely get together and talk...When we do have staff meetings, they are typically putting out the biggest fires then the smallest fires, rather than things that would address this particular topic.

P5

This challenge was compounded by *time constraints* as PCPs noted the difficulty in finding moments amid busy schedules to discuss referral practices or share insights from training:

In clinic we’re just always so busy. There aren’t really many opportunities for any major interprofessional collaboration. I mean, if you have a medical question or something, of course, yes, you can ask your colleague, but as far as just sharing information, usually the days are pretty jam-packed.

P6

#### Organizational Constraints

Second, PCPs described being limited by *organizational constraints*, particularly within large institutions where high specialization diminishes the perceived need to refine referral practices:

We’re at a large academic institution, things are so specialized that we just assume our patients are going to get very high-quality cancer care...often our main objective is to get our patients to the oncologist as quickly as possible.

P3

Institutional policies such as set referral practices further reduce the flexibility needed to refine these processes:

We do get pretty stuck here with referring. We have oncologists at [my institution] that we refer to and then if they feel they don’t have adequate options available, the oncologist usually refers the patient out [to another institution] which is right across the street from us. So, it is sort of a closed community.

P4

PCPs further expressed having *limited power* to make changes within their current roles, pointing to obstacles such as systemic barriers and hierarchical structures:

The health care system and the clinic schedule, in general, are very overwhelming, and there are a lot of burdens, as the PCP...So, I think it’s the system structure, in general, makes it difficult to make the changes, and it’s just not encouraging enough to really motivate me to make the changes.

P1

Another PCP cited hierarchical barriers as limiting their ability to effect change:

Because I’m a resident, this is a little bit trickier, honestly. I think when I’m attending, I’ll have more structural influence on how things are practiced.

P7

Finally, *fiscal policies* impacted PCPs’ ability to refine their referral practices:

Some of our referral practices are going to be based off what type of insurance [a patient has]. Or for us, like I mentioned at [my institution], our clinical decision-making is oftentimes within the realm of what [our institution] is willing to cover.

P4

## Discussion

### Principal Findings

This mixed methods pilot study provides a comprehensive evaluation of an innovative web-based training intervention aimed at enhancing PCPs’ knowledge, attitudes, communication, and practices regarding referrals to cancer care and CCTs. The Kirkpatrick evaluation model [46] provided a helpful framework for these evaluation findings. It is widely used in medical education to assess the outcomes of training and learning programs.

Level 1 of the Kirkpatrick evaluation model focuses on user reaction to the training intervention. Participants had high levels of satisfaction with the training intervention and reported high scores and positive comments about various aspects. Although satisfaction is important for participant engagement, it is not necessarily sufficient to produce desired changes.

Kirkpatrick level 2 focuses on learning as the next important outcome. Before the intervention, PCPs demonstrated relatively low levels of CCT knowledge, with a mean score of 55% (SD 17.9%) across 7 knowledge items, consistent with our earlier assessments of PCPs’ understanding of clinical trial concepts [40]. We found that PCPs’ knowledge about CCTs improved significantly after the intervention and was sustained at the 3-month follow-up, replicating what our team found in a prior study of PCPs in the New York City area [40]. This improvement is particularly important given PCPs’ previously self-identified limitations in cancer-specific knowledge [33,41]. Our evaluation also found an improvement in PCPs’ self-reported attitudes and beliefs about their role in working with patients with cancer, which was sustained at the 3-month follow-up, as well as willingness to communicate with their patients about CCTs, although this willingness to communicate was not sustained at the 3-month follow-up. However, willingness may be less important than actual behaviors.

With a foundation of change in knowledge, attitudes, and behavioral intentions, Kirkpatrick level 3 examines the impact of the educational intervention on behavior. According to our survey data, PCPs reported using the communication skills taught in the course with a higher percentage of patients about cancer treatment and CCTs at the 3-month follow-up than they did before the training within the context of making an initial referral for cancer care. Of the 7 communication items on the PRCP, only 1 (14%)—*emphasize partnership as patients go through cancer care*—did not show an improvement in scores*.* There may have been a ceiling effect on this item because the baseline score was already high. With respect to communication skills with patients who had already met with an oncologist, only 2 (29%) of the 7 items on the PRCP saw significant increases, and these 2 items focused specifically on the discussion of CCTs as a potential treatment option. It may be that the other items are not as relevant for a patient who has already had their initial meeting with the oncologist.

Another type of behavior we assessed was participants’ willingness to change their referral behavior after the training and the reports of these behavior changes at the 3-month follow-up. As these are slightly different concepts, we did not compare them; however, it was not unexpected to see lower scores on actual behavior change than intention.

Overall, the qualitative data underscored the positive impact that the training intervention had on the participating PCPs in terms of their evaluation of the intervention, their learning, and their behavior change. The qualitative data highlighted ways in which the intervention facilitated a more informed and patient-centered approach to care by increasing PCPs’ self-efficacy and confidence.

In addition to these positive outcomes, PCPs expressed some barriers to changing their CCT communication behaviors. They voiced concerns about their perceived lack of cancer expertise despite the course addressing the boundary of what PCPs should be expected to discuss. In our prior work, PCPs expressed a similar reluctance to discuss cancer treatments with patients [40], further highlighting a need to expand education regarding the role of PCPs in encouraging patients to engage in decision-making about their cancer treatment, including the consideration of CCTs. Importantly, the training does not suggest that PCPs determine patient eligibility for specific trials but rather prepares them to initiate supportive, patient-centered conversations that help patients feel empowered to ask questions and engage in screening discussions with their oncology team. PCPs can also support patients by reviewing laboratory or imaging results, helping to translate complex findings into accessible language, and explaining how these may relate to cancer treatment decisions or clinical trial eligibility without making determinations about eligibility themselves.

PCPs also relayed broader systemic challenges in their work settings that hindered making behavioral changes. The interview data helped to explain the lower scores on the willingness to change referral practices, as PCPs discussed the systematic barriers hindering collaborative efforts among health care professionals; these barriers in turn hindered their ability to integrate new knowledge and practices into their clinical settings.

### Implications for Practice

Our findings underscore the need for ongoing education to address gaps in the PCPs’ knowledge and skills needed to effectively communicate with patients about the potential of participating in CCTs as a high-quality treatment option. The evaluation of this educational intervention suggests that sustained change in knowledge, attitudes, and behavior can be achieved through an engaging, self-directed web-based intervention. As this training intervention is easily scalable, further dissemination may be able to positively impact quality of care and participation in clinical trials.

### Limitations and Future Research

As this was a pilot study, the sample size was small, although not too small to have enough power to detect significant results. Notably, the majority of the participants (n=17, 59%) were trainees working in academic settings, which may influence the generalizability of findings to the broader population of practicing PCPs. Furthermore, their receptiveness to the training and self-reported outcomes may not fully reflect the attitudes and behaviors of more experienced PCPs. Future research using a larger and more diverse sample of practitioners, including both trainees and practicing PCPs, would allow us to look for differences in evaluation metrics among different types of PCPs (residents, practicing physicians, physician assistants, and NPs). This study is also limited by its reliance on self-reported measures. Future research could incorporate patient interviews to provide additional insights into the effectiveness of the intervention from the patients’ perspectives. Using larger, more diverse samples to validate the intervention’s effectiveness across different practice settings and demographics is also recommended. Furthermore, including more follow-up surveys (eg, at 6 mo and 12 mo) could evaluate the sustained impact of educational interventions on PCPs’ knowledge retention and clinical practices over time. While this study identified several systemic barriers that may hinder CCT recruitment, addressing these challenges was beyond the scope of this pilot intervention. Future research should explore multilevel strategies to overcome these barriers and support sustained changes in clinical practice. This study, while focused on oncology referrals, is intended to be proof of concept for an intervention that can be adapted for other disease areas requiring specialist referrals, such as cardiology, rheumatology, and gastroenterology.

### Conclusions

This pilot study found that a self-guided, 1-hour web-based training intervention for PCPs improved their knowledge about CCTs as well as their attitudes and beliefs regarding their role in discussing treatment options with patients. The intervention also enhanced PCPs’ ability to communicate with patients about CCTs and prepare them for subsequent steps in the referral process, including discussions with specialists. Further testing of the training intervention in a larger sample can lead to future dissemination, with the potential to improve CCT accrual, especially among underrepresented groups. Furthermore, this study underscores the effectiveness of targeted educational interventions in equipping PCPs with the knowledge and confidence to communicate with patients about the potential of trial participation as a high-quality treatment option.

## Appendix

Multimedia Appendix 1 Full surveys, including demographic questions.

## Abbreviations

CCT: cancer clinical trial
NP: nurse practitioner
PCP: primary care provider
PGY: postgraduate year
SQUIRE 2.0: Standards for Quality Improvement Reporting Excellence
