# Supplementary material for: A Web-Based Training Intervention for Primary Care Providers on Preparing Patients for Cancer Treatment Decisions and Conversations About Clinical Trials: Evaluation of a Pilot Study Using Mixed Methods and Follow-Up
Source: JMIR Med Educ. 2025 Jul 17;11:e66892. doi: 10.2196/66892 (PMC12314468; doi:10.2196/66892)
Supplement: Multimedia Appendix 1 [file mededu_v11i1e66892_app1.docx]

**Measure 1:** Participant Characteristics. A 10-item survey that asks about participants’ demographics, professional background, and current professional setting.

1. Please indicate which degree you have earned.

1= MD

2= DO

3= Masters (PA Studies)

4= Masters (MSN)

1. Are you a resident or fellow?

1=No

2=Yes

1. What year of your training are you in?

1= PGY2

2= PGY3

3= PGY4

4= PGY5

5= Other (With a blank)

1. Choose one or more races that you consider yourself to be:

1= White

2= Black or African American

3= American Indian or Alaska Native

4= Asian

5= Native Hawaiian or Pacific Islander

6= Other (with a blank)

7= Prefer not to respond

1. Are you Hispanic or Latino or none of these?

1= Yes

2= None of these

3= Prefer not to respond

1. Do you describe yourself as a man, a woman, or in some other way?

1= A man

2= A woman

3= In some other way

4= Prefer not to respond

1. Please indicate what type of medical practice you work or train in.

1= Family practice

2= Internal medicine

3= Obstetrics and Gynecology

` 4= Other (please specify)

5= Geriatric medicine

1. In what kind of setting do you primarily practice or train?

1= Community-based independent private practice, not owned by a hospital and/or larger entity

2= Community-based practice owned by a large, non-hospital entity

3= Community-based practice owned by an academic medical center

4= Community hospital

5= Academic-affiliated hospital

6= Other (please describe)

1. Is your practice a designated Federally Qualified Health Center (FQHC)?

1= Yes
2= No

1. Is your service supported by the Indian Health Service?

1=Yes

2-No

**Measure 2: Knowledge of CCTs (KCCT).** A 7-item measure comprising true/false statements about cancer clinical trials.

Please indicate if you think the following statements about cancer treatment trials are true or false.

1. In a cancer treatment trial, patients will receive a placebo alone or the new treatment being tested.
   1. 1 = True
   2. 2 = False
   3. 3 = Don't Know
2. While about 25% of U.S. adults with cancer are eligible to participate in cancer treatment trials, only about 8% participate.
   1. 1 = True
   2. 2 = False
   3. 3 = Don't Know
3. Patients determined to be eligible for cancer treatment trials are usually offered the opportunity to participate.
   1. 1 = True
   2. 2 = False
   3. 3 = Don't Know
4. Patients from racial and ethnic minority groups eligible to participate in cancer treatment trials tend to be offered the opportunity to participate less frequently than are white patients.
   1. 1 = True
   2. 2 = False
   3. 3 = Don't Know
5. When offered the opportunity to participate in a cancer treatment trial, patients from racial and ethnic minority groups agree to participate at about the same rate as do white patients.
   1. 1 = True
   2. 2 = False
   3. 3 = Don't Know
6. Recommendation by a trusted physician is a primary factor influencing a patient’s decision to enroll in a cancer treatment trial.
   1. 1 = True
   2. 2 = False
   3. 3 = Don't Know
7. Federal law now requires private insurers, Medicare and Medicaid to cover routine patient care costs in most cancer treatment trials.
   1. 1 = True
   2. 2 = False
   3. 3 = Don't Know

**Measure 3: Cancer Clinical Trials Attitudes and Beliefs (CCTAB).** A 4-item Likert-type measure that assesses attitudes, beliefs, and behavioral intentions related to cancer clinical trials.

On a scale of “Strongly Disagree” to “Strongly Agree,” please indicate your level of agreement with the following statements.

1. Health care providers like me have an important role in educating patients that there is often more than one option for treatment, before the referral to a specialist.
   1. 5 = Strongly Agree
   2. 4 = Agree
   3. 3 = Neither agree nor disagree
   4. 2 = Disagree
   5. 1 = Strongly Disagree
2. Health care providers like me have an important role in educating patients about the possibility of receiving cancer treatment through a clinical trial, before the referral to a specialist.
   1. 5 = Strongly Agree
   2. 4 = Agree
   3. 3 = Neither agree nor disagree
   4. 2 = Disagree
   5. 1 = Strongly Disagree
3. Health care providers like me have an important role in supporting patients’ decision to participate in a cancer treatment trial.
   1. 5 = Strongly Agree
   2. 4 = Agree
   3. 3 = Neither agree nor disagree
   4. 2 = Disagree
   5. 1 = Strongly Disagree
4. Health care providers like me can make a difference in the quality of cancer care our patients receive.
   1. 5 = Strongly Agree
   2. 4 = Agree
   3. 3 = Neither agree nor disagree
   4. 2 = Disagree
   5. 1 = Strongly Disagree

**Measure 4: Patient Communication and Referral Practices (PCRP)**. A 14-item measure that assesses PCPs’ communication and referral behaviors before (7 questions) and after (7 questions) returning patients’ initial referral to a cancer specialist to discuss treatment options.

*For patients diagnosed with cancer in the past three months, prior to making a referral, approximately what percentage of patients did you:*

1. Explore concerns about cancer treatment in general?
2. Educate about cancer treatment in general?
3. Educate about receiving treatment with a cancer clinical trial?
4. Encourage inquiry about treatment options?
5. Encourage inquiry about receiving treatment through a cancer clinical trial?
6. Engage in helping patients plan these inquiries with a specialist?
7. Emphasize partnership as patients go through cancer care?

*For patients diagnosed with cancer in the past three months, after patients returned following their initial referral, approximately what percentage of patients did you:*

1. Explore concerns about cancer treatment in general?
2. Educate about cancer treatment in general?
3. Educate about receiving treatment with a cancer clinical trial?
4. Encourage inquiry about treatment options?
5. Encourage inquiry about receiving treatment through a cancer clinical trial?
6. Engage in helping patients plan these inquiries with a specialist?
7. Emphasize partnership as patients go through cancer care?

**Measure 5: Willingness to change (WTCH)**. A 3-item Likert-type measure that focuses on PCP’s willingness to make changes in their practice

On a scale of “Strongly Disagree” to “Strongly Agree,” please indicate your level of agreement with the following statements.

*Post survey*

1. I am willing to make needed changes to whom I refer my patients for cancer care, in order to improve their access to cancer treatment trials.
2. I am willing to talk with colleagues in my practice about needed changes in how we educate our patients with cancer prior to referral.
3. I am willing to talk with colleagues in my practice about needed changes to whom we refer our patients for cancer care, in order to improve their access to cancer treatment trials.

*3-Month follow-up survey*

1. I have made changes to whom I refer my patients for cancer care, in order to improve their access to cancer treatment trials.
2. I have talked with colleagues in my practice about needed changes in how we educate our patients with cancer prior to referral.
3. I have talked with colleagues in my practice about needed changes to whom we refer our patients for cancer care, in order to improve their access to cancer treatment trials.

**Measure 6: Willingness to Communicate (WTCO)**. A 6-item Likert-type measure that focuses on PCP’s willingness to engage with patients who have a cancer diagnosis.

Once a patient has received a cancer diagnosis, many PCPs make the initial referral to a cancer specialist. The following questions are about your willingness to engage with these patients PRIOR to that referral.

1. I am willing to explore patients' concerns about cancer treatment.
2. I am willing to educate these patients that there is often more than one option for treatment.
3. I am willing to educate these patients about the possibility of receiving treatment within a cancer clinical trial.
4. I am willing to encourage these patients to ask questions of the specialist about different treatment options.
5. I am willing to encourage these patients to ask questions of the specialist about receiving treatment within a cancer clinical trial.
6. I am willing to emphasize my role as a partner throughout these patients’ cancer care.

**Measure 7: Intervention Usability**. A 5-item survey that asks about participants’ device (e.g., computer tablet), browser type, and if they encountered any technical issues while taking the online course.

1. What did you use to participate in the course? (Please select all that apply)

1= Computer (laptop or desktop)

2= Tablet (iPad or other)

3= Smart phone

4= Other

1. What type of Internet browser did you use to participate in the course? (Please select all that apply)

1= Google Chrome

2= Safari

4= Firefox

5= Microsoft Edge

6= Other (with a blank)

7= I’m not sure

1. Please rate how clear it was to progress through the four modules.

1= Extremely unclear

2= Somewhat unclear

3= Neither clear nor unclear

4= Somewhat clear

5= Extremely clear

1. Did you encounter any problems navigating the course?

1= Yes

2= No

1. Please describe the problem(s) you encountered navigating the course.

1=Asked if UQ4=1
2=Open ended

**Measure 8: Overall Course Satisfaction.** A 10-item Likert-type measure where participants evaluate their training experience (e.g., content, logistics) and intervention acceptability, along with two open-ended questions for additional feedback.

*Please rate your agreement with the following statements*

1. The course presented information in a clear and organized manner.
2. The length of the course was appropriate.
3. Course content was relevant to my work.
4. The course scenarios facilitated my understanding.
5. The graphics (graphs, pictures, illustrations) added to the effectiveness of the presentation.
6. The interactive features in the course activities helped me learn.
7. The right amount of information was provided.
8. The course provided an adequate evidence base to support the content.
9. The scenarios shown in the course felt authentic.
10. The clinical interactions shown in the course were relatable to me.
